# Literature review on shift work and nurse’s burnout

**DOI:** 10.1080/07853890.2021.1897458

**Published:** 2021-09-28

**Authors:** João Reisinho, Rita Rodrigues, António Fernandes, João Sardinha, Pedro Santos, Daniel Sousa, Fernanda Loureiro, Vanessa Antunes

**Affiliations:** a Nursing Student, Escola Superior de Saúde Egas Moniz, Caparica, Portugal

## Abstract

**Introduction:**

In Europe 20% of the working population is somehow involved in shift work. Studies show that it can have a negative impact on workers' health and well-being, with direct consequences on performance and efficiency [1]. Nurses are among health professionals with a higher risk of burnout [2], which can negatively affect their alertness, attention and concentration, with direct consequences on patient’s health [3]. The aim of this study is to identify relevant factors regarding the impact of shift work in nurses’ health and well-being.

**Materials and methods:**

This study is a literature review which consists of a narrative and comprehensive analysis of the literature [4]. Articles were search in the following databases: Academic Google, Scientific Electronic Library Onine (SciELO) and EBSCO Host. Keywords were defined according to the acronym PCC (Population, Concept, Context): P: nurs*; C: burnout; C: shift work*). Inclusion criteria were: articles in Portuguese and English, available in full-text, published between 2013 and 2018. The review was conducted in parallel by two independent researchers. 10 articles were selected for final review. Data were extracted and synthesised using an information systematisation table.

**Results:**

The articles allowed to identify the impact of shift work on nurses physical (interrupts the circadian rhythm, affects sleep quality, causes fatigue, gastrointestinal, neuropsychological, cardiovascular, and musculoskeletal disorders), mental (depersonalization, cynicism, aggression and frustration), and social dimensions (deterioration of family relationships and social life). Although most studies mention the impact of burnout on nurses professional performance and quality of care, no details are provided on the specific consequences for healthcare consumers, or for the health system itself.

**Discussion and conclusions:**

Although there’s a widespread concern about the impact of shift work on nurse’s health, few studies mention the consequences of burnout on nurse’s performance and on the quality of care. Strategies to minimise the impact of burnout are superficially addressed and reduced to the intervention of occupational health [5]. Developing, testing, and implementing intervention programs to reduce burnout may produce a variety of beneficial effects. Burnout appears to be an important indicator for healthcare leaders at both strategical and tactical level, to track and solve quality of care issues particularly in the current context of nursing shortage [6].
